# Machine learning prediction of pathological complete response and overall survival of breast cancer patients in an underserved inner-city population

**DOI:** 10.1186/s13058-023-01762-w

**Published:** 2024-01-10

**Authors:** Kevin Dell’Aquila, Abhinav Vadlamani, Takouhie Maldjian, Susan Fineberg, Anna Eligulashvili, Julie Chung, Richard Adam, Laura Hodges, Wei Hou, Della Makower, Tim Q. Duong

**Affiliations:** 1 Department of Radiology, Montefiore Health System and Albert Einstein College of Medicine, 111 E 210th St, Bronx, NY 10467 USA; 2 Department of Pathology, Montefiore Health System and Albert Einstein College of Medicine, Bronx, NY USA; 3 Department of Oncology, Montefiore Health System and Albert Einstein College of Medicine, Bronx, NY USA; 4 Center for Health Data Innovation, Montefiore Health System and Albert Einstein College of Medicine, Bronx, NY USA

**Keywords:** Molecular subtypes, Tumor subtypes, Socioeconomic status, Progression-free survival, Deep learning, Health disparity

## Abstract

**Background:**

Generalizability of predictive models for pathological complete response (pCR) and overall survival (OS) in breast cancer patients requires diverse datasets. This study employed four machine learning models to predict pCR and OS up to 7.5 years using data from a diverse and underserved inner-city population.

**Methods:**

Demographics, staging, tumor subtypes, income, insurance status, and data from radiology reports were obtained from 475 breast cancer patients on neoadjuvant chemotherapy in an inner-city health system (01/01/2012 to 12/31/2021). Logistic regression, Neural Network, Random Forest, and Gradient Boosted Regression models were used to predict outcomes (pCR and OS) with fivefold cross validation.

**Results:**

pCR was not associated with age, race, ethnicity, tumor staging, Nottingham grade, income, and insurance status (*p* > 0.05). ER−/HER2+ showed the highest pCR rate, followed by triple negative, ER+/HER2+, and ER+/HER2− (all *p* < 0.05), tumor size (*p* < 0.003) and background parenchymal enhancement (BPE) (*p* < 0.01). Machine learning models ranked ER+/HER2−, ER−/HER2+, tumor size, and BPE as top predictors of pCR (AUC = 0.74–0.76). OS was associated with race, pCR status, tumor subtype, and insurance status (*p* < 0.05), but not ethnicity and incomes (*p* > 0.05). Machine learning models ranked tumor stage, pCR, nodal stage, and triple-negative subtype as top predictors of OS (AUC = 0.83–0.85). When grouping race and ethnicity by tumor subtypes, neither OS nor pCR were different due to race and ethnicity for each tumor subtype (*p* > 0.05).

**Conclusion:**

Tumor subtypes and imaging characteristics were top predictors of pCR in our inner-city population. Insurance status, race, tumor subtypes and pCR were associated with OS. Machine learning models accurately predicted pCR and OS.

**Supplementary Information:**

The online version contains supplementary material available at 10.1186/s13058-023-01762-w.

## Introduction

Breast cancer is a complex disease with highly heterogeneous tumor characteristics and clinicopathological profiles [1]. Predicting response to neoadjuvant chemotherapy and overall survival in breast cancer patients remains a crucial challenge for disease management. In addition, racial, ethnic, and socioeconomic disparities could also influence breast cancer outcomes [2, 3], highlighting the need for diverse and inclusive datasets to develop more accurate predictive models.

Molecular subtypes of breast cancer exhibit distinct clinicopathological profiles [4]. These subtypes have varying responses to different treatment modalities, emphasizing the importance of tailoring therapy based on tumor subtype [5, 6]. Incorporating molecular subtype information into predictive models helps better predict treatment response and overall survival, guiding clinicians in making informed decisions. Racial and ethnic groups differ in their prevalence of tumor subtypes, which could contribute to inconsistent prognoses [7]. Most breast cancer clinical trials also lack racial and ethnic diversity, with Blacks and Hispanics largely underrepresented, presenting a barrier to precision medicine for these populations [7, 8]. Moreover, socioeconomic status could also affect outcomes.

Tumor characteristics, clinicopathological profiles, patient profiles, and other variables interact, making it challenging to identify independent risk factors that predict outcomes. Recent advancements in machine learning predictive modeling have shown promise in addressing this challenge [9, 10] because machine learning can deal with complex datasets without the need to specify a priori the complex relationship among the large number of variables. These models leverage algorithms that learn patterns from a vast array of patient data [9, 10], including demographic information, histopathological features, treatment regimens, molecular profiles, and socioeconomic factors. By harnessing the power of machine learning, robust and accurate models that integrate diverse populations and tumor subtypes can be developed, aiding in personalized medicine for breast cancer patients. However, machine learning also has the potential to exacerbate racial and ethnic disparities with imbalanced representation of demographics [11].

Pathologic complete response (pCR) serves as a surrogate marker for neoadjuvant treatment efficacy in breast cancer patients [12–14]. Achieving pCR, defined as the absence of invasive carcinoma in the breast and axillary lymph nodes following neoadjuvant treatment, is associated with improved overall survival (OS) [12–14]. Accurate prediction of pCR can guide treatment decisions, potentially sparing patients from unnecessary interventions or identifying those who may require additional therapies [15]. Overall survival reflects the long-term outcomes and effectiveness of treatment strategies [16]. Identifying predictors of OS can assist in tailoring escalating therapy or follow-up intervals toward discrete risk factors. Machine learning predictive models offer the potential to integrate large number of clinical, pathological, molecular data and socioeconomic factors to provide personalized treatments to improve pCR and OS for individual patients [17].

The goals of this study were to employ four machine learning models to identify key risk factors among a large array of clinicopathological, tumor subtypes, insurance status, income, and imaging characteristics from a diverse racial, ethnic, and socioeconomic status and to predict pCR and OS at 7.5 years after diagnosis in breast cancer patients. Four machine learning models were employed to predict pCR and 7.5-year OS.

## Methods

### Data sources

This retrospective study was approved by our IRB (institution removed for blinded review but can be identified if needed) with waived informed consent (2020-12169). The study followed the Strengthening the Reporting of Observational Studies in Epidemiology (STROBE) reporting guidelines. The patient cohort comprised of all patients diagnosed with invasive breast cancer within our institution’s health system which serves an inner-city urban population between 01/01/2012 and 12/31/2021 and treated with neoadjuvant chemotherapy followed by surgery in our health system. Data were obtained from the cancer registry of our institution and via chart review of the electronic medical records and radiology reports. There were 509 patients and 34 patients excluded due to missing pCR outcome, with a final sample size of 475. Missing non-MRI data averaged 3.1%. Patients with missing data were excluded from ML modeling. Only 240 patients had MRI reports describing all relevant imaging elements. The sample size for each analysis is provided in respective tables and figures.

The clinicopathological data included age, race (White, Black, Asian, others), ethnicity (Hispanic, non-Hispanic), clinical tumor (T) and nodal (N) stage by TNM staging, Nottingham grade (Nottingham Grade 1 (well differentiated), 2 (moderately differentiated), 3 (poorly differentiated)), tumor subtypes (ER−/HER2+, ER+/HER2+, ER+/HER2−, and triple negative), and radiological data from MRI radiology reports (background parenchymal enhancement, tumor size, multifocal lesions, skin involvement, satellite lesions, pectoralis muscle involvement, lymph node involvement, chest wall involvement, nipple involvement, and multicentric lesions). In addition, income quintiles and insurance status were also tabulated. The primary outcomes were pCR and OS.

### Logistic regression

Logistic regression was performed to compute the odds ratios (ORs) of risk factors associated with outcomes (*N* = 475). Inputs for pCR ORs included demographics, tumor subtypes, insurance status, and income quintile. Inputs for overall survival ORs included demographics, tumor subtypes, insurance status, income quintile, and pCR status. Insurance status included private, Medicaid, Medicare. Self-pay and uninsured status amounted to < 1% of sample size and were not included in OR calculation.

### Predicting pCR and OS

Four predictive models, Logistic Regression, Neural Network (NN), Random Forest (RF), and Gradient Boosted Regressor (GBR), were created to predict pCR in patients who received neoadjuvant chemotherapy.

Multivariate logistic regression was used as a baseline for comparison. The solver, or the algorithm used by the LR model for optimization, was newton-cg which uses the second-order Taylor Series to create an approximation for gradient optimization [18].

For Neural Networks we used a fully connected feed-forward neural network with one hidden layer and one output layer [19]. The hidden layer contains 32 neurons, activation function of ReLU, and l2 regularization with regularization factor of 0.01. The NN utilizes a mean squared error loss function and Adam optimizer with a learning rate of 0.01.

A Random Forest Algorithm was utilized with a max depth of 1 for the univariate analysis, and max depth of 10 for multivariate analysis to limit overfitting [20]. The algorithm creates multiple decision trees to create a more holistic and better result when it comes to multivariate analysis.

Gradient Boosted Regression utilizes the Boosting ensembling technique which combines multiple weak learners, which in this case is a regression model, and ensembles them together to create a strong learner, or a stronger regression model [21]. In our model, we utilized a max depth of 1, 50 estimators, and a learning rate of 0.001 for the univariate analysis, and a max depth of 3, 100 estimators, and a learning rate of 0.1.

Hyperparameter tuning was conducted using the grid search method. For the neural networks, the grid search algorithm combined powers of 2 for the number of neurons and powers of 10 for the learning rate. For the Random Forest, the grid search algorithm combined numbers from 1 to 50 for the max depth. For the Gradient Boosted Regression algorithm, the grid search algorithm combined numbers from 1 to 100 for the depth and estimators and powers of 10 for the learning rate.

These analyses were conducted using Python, specifically the TensorFlow library for the neural networks and the sklearn library for RF, Logistic Regression, and GBR models. An 80/20 train validation split was utilized with fivefold cross validation [22, 23]. Performance metrics (such as AUCs) were reported for test (validation) sets only using fivefold cross validation from which mean ± SD were obtained. A 50% probability threshold was used to calculate sensitivity/specificity. 95% confidence interval was chosen.

Data used to predict pCR included demographics, clinical staging, tumor subtypes, and MRI data. Data used to predict OS included demographics, tumor subtypes, clinical staging, tumor subtypes, MRI data, and pCR status. OS was determined to be the proportion of patients alive 7.5 years after diagnosis. Insurance status and income quintiles were not used. The top 10 predictors were identified and used to evaluate performance indices.

### Kaplan–Meier survival analysis

Kaplan–Meier survival analysis for patients with breast cancer was performed with stratification of pCR status, tumor subtypes, insurance, race, ethnicity, and income quintiles. For race and ethnicity, outcomes were also sub-stratified by pCR status.

### Statistical analysis

*Χ*^2^ tests were performed using R Studio (version 3.1). Logistic regression analysis used R studio or Python (version 3.10.9) for identifying risk factors and for predicting outcomes. Hazard ratios were obtained using Cox-regression analysis using R studio and Kaplan–Meier curves were generated using Python. ANOVA was used for comparison with three or more groups. A *p* < 0.05 was used to indicate statistical significance unless otherwise specified.

## Results

### pCR

Patient profiles stratified by pCR status are summarized in Table 1. pCR was not significantly associated with age (≥ 50yo vs < 50yo (*p* = 0.20), race (*p* = 0.87) or ethnicity (*p* = 1.0), T-stage (*p* = 0.09), N stage (*p* = 0.31), and Nottingham grade (*p* = 0.09), but was significantly associated with tumor subtype (*p* < 0.001), with ER−/HER2+ (56.5%) having the highest pCR rate, followed by triple negative (31.0%), ER+/HER2+ (23.0%) and ER+/HER2− (8.5%).

**Table 1:** Patient profiles by pCR status (*N* = 475)

Patient characteristics	*N* (%)	% pCR	% no pCR	*p* value
All patients with pCR data	475 (100%)	27.4%	72.6%	–
Age, years (median 55.7 [IQR 17.57])				0.20
≥ 50	319 (67.2%)	25.4%	74.6%	
< 50	156 (32.8%)	31.4%	68.6%	
Race (*n*, %)				0.87
Hispanic white	96 (20.2%%)	25.0%	75.0%	
Non-Hispanic white	64 (13.5%)	21.9%	78.1%	
Black	263 (55.4%)	28.5%	71.5%	
Asian	22 (4.6%)	31.8%	68.2%	
Other	30 (6.3%)	33.3%	66.7%	
Ethnicity (*n*, %)				1.0
Hispanic	164 (34.5%)	27.4%	72.6%	
Not Hispanic	311 (65.5%)	27.3%	72.7%	
Clinical T-stage (*n*, %)				0.09
T1	66 (14.0%)	30.0%	70.0%	
T2	240 (51.1%)	31.0%	69.0%	
T3 and T4	176 (34.9%)	21.3%	78.7%	
Clinical N Stage (*n*, %)				0.31
N0	157 (33.0%)	32.5%	67.5%	
N1	243 (51.2%)	24.7%	75.3%	
N2	37 (7.8%)	21.6%	78.4%	
N3	38 (8.0%)	28.9%	71.1%	
Nottingham grade (*n*, %)				0.09
Grade 1	5 (1.2%)	0.0%	100.0%	
Grade 2	129 (31.5%)	21.7%	78.3%	
Grade 3	276 (67.3%)	29.7%	70.3%	
Tumor subtype groups (*n*, %)				< 0.001
ER−/HER2+ (± PR+)	69 (15.1%)	56.5%	43.5%	
ER+/HER2+ (± PR+)	87 (19.0%)	23.0%	77.0%	
ER+/HER2− (± PR+)	130 (28.4%)	8.5%	91.5%	
ER−/HER2− (PR−) (triple negative)	171 (37.4%)	31.0%	69.0%	
BPE pre-NAC (*n*, %)				0.009
Mild	163 (63.0%)	35.6%	64.4%	
Moderate	83 (32.0%)	24.1%	75.9%	
Marked	13 (5.0%)	0.0%	100.0%	
MRI tumor size (LD, cm) (n, %)				0.003
Small (≤ 2)	38 (15.8%)	34.2%	65.8%	
Medium (> 2 to 5)	148 (61.7%)	35.1%	64.9%	
Large (> 5)	54 (22.5%)	11.1%	88.9%	
Annual income				0.77
First quintile ($21,846.00–$34.860.00)	87 (21.5%)	33.3%	66.7%	
Second quintile ($34,860.00–$42,639.00)	100 (24.7%)	25.0%	75.0%	
Third quintile ($42,639.00–$58,814.20)	55 (13.6%)	29.1%	70.9%	
Fourth quintile ($58,814.20–$70,107.00)	81 (20.0%)	27.2%	72.8%	
Fifth quintile ($70,107.00–$218,493.00)	82 (20.2%)	30.5%	69.5%	
Insurance type				0.58
Private	139 (34.5%)	28.1%	71.9%	
Medicaid	209 (51.9%)	30.6%	69.4%	
Medicare	51 (12.7%)	23.5%	76.5%	
Uninsured	4 (1.0%)	50%	50.0%	

*p* values indicate comparison between pCR versus non-pCR by *χ*^2^ test

pCR was significantly associated with tumor size (*p* = 0.003), with tumors > 5 cm having a lower pCR rate (11.1%) compared with tumors measuring ≤ 2 cm and 2–5 cm (34.2% and 35.1%, respectively). Mild BPE had the highest rate of pCR (35.6%) followed by moderate (24.1%) and marked (0.0%) (*p* < 0.03) BPE. This was unexpected and we further investigated and found that patients with marked BPE consisted of mostly of ER+/HER2− and ER+/HER2+, and larger tumor size, and poorer differentiation. Income and insurance status were not significantly associated with pCR (*p* > 0.05).

Table 2 shows the composition and pCR for different race and ethnicity grouped by tumor subtypes. Blacks had higher composition of triple negative (*p* < 0.05), lower composition of the ER+/HER2+ and ER+/HER2− subtypes. There was however no significant difference due to race (*p* > 0.05) nor ethnicity (*p* > 0.05) for each tumor subtype. Note that there were high proportion of HER2 positive breast cancers and relatively low proportion of ER/PR positive cases because ER/PR positive patients are known to be less responsive to neoadjuvant chemotherapy and they were not given neoadjuvant chemotherapy. When data were modeled individually for each of the four tumor subtype groups (Additional file 1: Table S1), radiographic tumor size and BPE were highly ranked among predictors, but T-stage ranked lower as a predictor of pCR.

**Table 2:** Percent of patients and pCR for race and ethnicity grouped by molecular subtypes (*N* = 475), **(B)** AUCs for all four univariate models across all 4 tumor subtypes

	ER−/HER2+	ER+/HER2+	ER+/HER2−	TN
Percent of patients				
White	18 (11%)	39 (24%)	51 (32%)	49 (31%)
Black	42 (16%)	39 (15%)^a^	61 (23%)^a^	109 (41%)^a^
Percent with pCR				
White	9 (50%)	9 (23%)	6 (12%)^b^	14 (29%)
Black	23 (55%)	8 (21%)^b^	4 (7%)^b^	34 (31%)^b^
Percent of patients				
Hispanic	24 (14%)	33 (17%)	54 (24%)	48 (40%)
Non-Hispanic	45 (14%)	54 (17%)	76 (24%)	123 (40%)
Percent with pCR				
Hispanic	14 (58%)	6 (18%)^b^	7 (13%)^b^	17 (35%)
Non-Hispanic	25 (55%)	14 (26%)	4 (5%)^b^	36 (29%)

Note that percentages do not add up to 100% because “other” race due to small sample sizes were not included

^a^Indicates *p* < 0.05 between race or ethnicity

^b^Indicates *p* < 0.05 different from ER−/HER2+

Table 3 shows the odds ratios for achieving pCR. Race (*p* > 0.05) and ethnicity (*p* > 0.05) did not contribute to different odds of achieving pCR. ER+/HER2− had the lowest likelihood of achieving pCR (OR = 0.085, [0.037,0.194], *p* < 0.0001), followed by triple negative (OR = 0.406, [0.219,0.754], *p* = 0.004), and ER+/HER2+ (OR = 0.285, [0.137,0.593] *p* < 0.0001) compared to ER−/HER2+. Patients in different income quintiles, except with the 1st quintile, and patients with insurance status did not have lower odds of achieving pCR.

**Table 3:** Odds ratios for pCR as outcome for demographics, tumor subtypes, pCR, income quintile, and insurance status (*N* = 433 out of 475)

(A) Odds ratio for pCR as the outcome	OR	2.5%	97.5%	*p* value
Race (white as reference)				
Black versus non-Hispanic White (ref)	1.515	0.737	3.114	0.259
Asian versus non-Hispanic White (ref)	2.207	0.898	5.425	0.085
Ethnicity				
Hispanics	1.267	0.809	1.987	0.301
Subtypes (ER− and HER2+ as reference)				
Triple negative	0.406	0.219	0.754	0.004
ER+ and HER2+	0.285	0.137	0.593	< 0.0001
ER+ and HER2−	0.085	0.037	0.194	< 0.0001
Incomes (5th quintile (highest) as reference)				
4th	0.899	0.434	1.862	0.774
3rd	0.942	0.417	2.219	0.885
2nd	0.798	0.395	1.614	0.530
1st	1.204	0.608	2.382	0.001
Insurance status (private as reference)				
Medicaid	0.838	0.502	1.399	0.498
Medicare	0.724	0.342	1.533	0.399

Table 4 summarizes the results of four different ML models. All 4 models consistently ranked ER+/HER2−, ER−/HER2+, radiographic tumor size, and BPE as top predictors, but ER+/HER2+ and triple negative were not top predictors. Accuracy ranged from 0.697 to 0.731, specificity 0.736 to 0.890, sensitivity 0.555 to 0.799 and AUC 0.743 to 0.755.

**Table 4:** Multivariate analysis of top predictors of pCR *(N* = 240)

(B)	Predictors for pCR as the outcome	Accuracy	Sensitivity	Specificity	AUC
NN	ER+/HER2−, ER−/HER2+, TS, BPE, Mu, SI, Age, NG, Nstage, T-stage	0.731 ± 0.046	0.799 ± 0.092	0.7360 ± 0.09	0.7540 ± 0.039
RF	ER+/HER2−, ER−/HER2+, TS, BPE, Age, Mu, SI, NG, NS, T-stage	0.720 ± 0.056	0.583 ± 0.147	0.774 ± 0.068	0.7520 ± 0.064
LR	ER+/HER2−, ER−/HER2+, TS, BPE, Mu, SI, Age, NG, T-stage, Nstage	0.720 ± 0.075	0.555 ± 0.118	0.890 ± 0.052	0.7550 ± 0.043
GBR	ER+/HER2−, ER−/HER2+, TS, BPE, Age, Mu, SI, T-stage, NG, Nstage	0.697 ± 0.055	0.666 ± 0.209	0.785 ± 0.054	0.7430 ± 0.084

The performance metrics are mean ± standard deviation for four different models: Neural network (NN), random forest (RF), logistic regression (LR), and gradient boosted regressor (GBR)

*TS* tumor size, *BPE* background parenchymal enhancement, *Mu* multifocal, *SI* skin involvement, *NG* Nottingham grade

### Overall survival

**Fig. 1:**
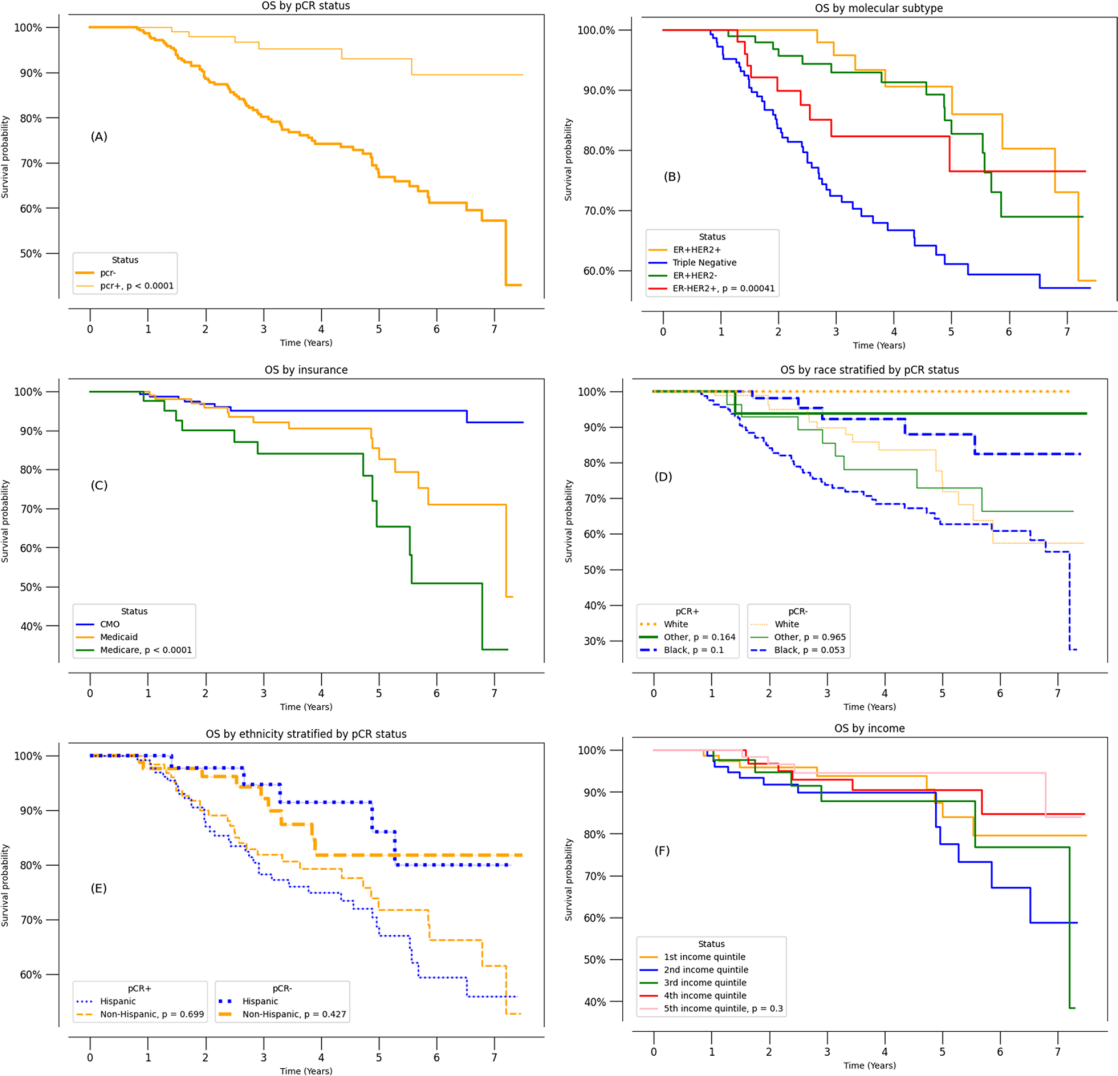
Kaplan–Meier survival curves for patients with breast cancer (*N* = 475) by pCR status, tumor subtypes, insurance status, race, ethnicity, and income. Patients belonging to Asian and “other” race (*n* = 19 and *n* = 26, respectively) were grouped together for comparison with white (*n* = 136) and Black (*n* = 233) races. The median time to last contact was 3.83 years (IQR: 2.13–6.46). All patients had followed up with a recorded date of last contact, among whom there were 85 events

Figure 1 shows the Kaplan–Meier survival analysis (*N* = 475). Patients who achieved pCR had a clear survival benefit compared to those who did not (HR = 0.1898, 95% CI (0.08275–0.4354), *p* < 0.0001) (Fig. 1A). Tumor subtype was significantly associated with survival probability (Fig. 1B). Patients with triple negative disease were significantly more likely to die than those with ER+/HER2+ (HR = 0.3109, 95% CI: [0.1468–0.6582], *p* = 0.0023), ER+/HER2− (HR = 0.4020, 95% CI: [0.2245–0.7198], *p* = 0.0022) and ER−/HER2+ (HR = 0.5077, 95% CI: [0.2487–1.0363], *p* = 0.0626). There were no significant differences in survival probability among ER−/HER2+, ER+/HER2+, and ER+/HER2− subtypes (*p* > 0.05). Insurance status was significantly associated with survival probability (Fig. 1C). Patients on Medicaid (HR = 3.29 95% CI: [1.39–7.77], *p* = 0.007) and Medicare (HR = 6.93 95% CI: [2.824–17.01], *p* < 0.0001) showed higher odds of mortality compared to those on private insurance. Note that patients on Medicare were significantly older (*p* < 0.05 ANOVA). There were no significant differences in survival probability by race (*p* > 0.05) and ethnicity (*p* > 0.05) when stratified by pCR (Fig. 1D, E**)**. There was no significant difference in survival probability income status (*p* > 0.05) (Fig. 1F).

Patient profiles for OS at 7.5 years after diagnosis are summarized in Table 5. OS was significantly lower for those who were ≥ 50yo compared to < 50yo (*p* = 0.04). OS was significantly associated with T-stage (*p* < 0.0001), N stage (*p* < 0.001), and race (*p* = 0.03), but not ethnicity (*p* = 0.53) or Nottingham grade (*p* = 0.92). OS was significantly associated with tumor subtype (*p* < 0.001), with triple negative having the lowest survival (72.5%). OS was not significantly associated with BPE (*p* = 0.89), tumor size (*p* = 0.15), and income (*p* = 0.39), whereas OS was significantly associated with pCR (*p* < 0.0001), and insurance status (*p* = 0.0002) by *χ*^2^ analysis.

**Table 5:** Patient profiles by OS status at 7.5 years (*N* = 475)

Patient characteristics	*N* (%)	% OS	% no OS	*p* value
All patients	475 (100%)	82.9	17.1	–
Age, years (median 55.7 [IQR 17.57])				0.04
≥ 50	319 (67.2%)	80.3	19.7	
< 50	156 (32.8%)	88.5	11.5	
Race (*n*, %)				0.03
Hispanic white	96 (20.2%)	86.5	13.5	
Non-Hispanic white	64 (13.4%)	93.8	6.2	
Black	263 (55.4%)	79.1	20.9	
Asian	22 (4.6%)	90.9	9.1	
Other	30 (6.3%)	76.7	23.3	
Ethnicity (*n*, %)				0.53
Hispanic	164 (34.5%)	84.8	15.2	
Not Hispanic	311 (65.5%)	82.0	18.0	
Clinical T-stage (*n*, %)				< 0.0001
T1	66 (14.0%)	89.4	10.6	
T2	240 (51.1%)	89.6	10.4	
T3 and T4	164 (34.9%)	71.3	28.7	
Clinical N Stage (*n*, %)				< 0.001
N0	157 (33.0%)	91.7	8.3	
N1	243 (51.2%)	81.1	18.9	
N2	37 (7.8%)	62.2	37.8	
N3	38 (8.0%)	81.6	18.4	
Nottingham grade (*n*, %)				0.92
Grade 1	5 (1.2%)	80.0	20.0	
Grade 2	129 (31.5%)	83.7	16.3	
Grade 3	276 (67.3%)	80.4	19.6	
Tumor subtype groups (n, %)				< 0.001
ER−/HER2+ (± PR+)	69 (15.1%)	87.0	13.0	
ER+/HER2+ (± PR+)	87 (19.0%)	90.8	9.2	
ER+/HER2− (± PR+)	130 (28.4%)	88.5	11.5	
ER−/HER2− (PR−) (triple negative)	171 (37.4%)	72.5	27.5	
BPE Pre-NAC (*n*, %)				0.89
Mild	163 (63.0%)	89.0	11.0	
Moderate	83 (32.0%)	88.0	12.0	
Marked	13 (5.0%)	92.3	7.7	
MRI tumor size (LD, cm) (*n*, %)				0.15
Small (≤ 2)	38 (15.8%)	92.1	7.9	
Medium (> 2 to 5)	148 (61.7%)	90.5	9.5	
Large (> 5)	54 (22.5%)	81.5	18.5	
PCR				< 0.0001
Yes	100 (22.5%)	95.4	4.6	
No	345 (77.5%)	78.3	21.7	
Annual income				0.39
First quintile ($21,846.00–$34.860.00)	87 (21.5%)	90.8	9.2	
Second quintile ($34,860.00–$42,639.00)	100 (24.7%)	87.0	13.0	
Third quintile ($42,639.00–$58,814.20)	55 (13.6%)	89.1	10.9	
Fourth quintile ($58,814.20–$70,107.00)	81 (20.0%)	92.6	7.4	
Fifth quintile ($70,107.00–$218,493.00)	82 (20.2%)	95.1	4.9	
Insurance type				0.0002
Private	139 (34.5%)	96.2	3.8	
Medicaid	209 (51.9%)	88.5	11.5	
Medicare	51 (12.6%)	76.5	23.5	
Uninsured	4 (1.0%)	100.0	0.0	

*p* values indicate comparison between OS versus non-OS (7.5 years). Note that only 4 patients were uninsured and were included in the insurance status section. Note there was no missing OS data

Table 6 shows OS at 7.5 years for different races and ethnicities grouped by tumor subtypes. Blacks vs whites showed no differences in OS for any subtypes (*p* > 0.05). Hispanics vs non-Hispanics also showed no differences in OS across any subtypes (*p* > 0.05); however, non-Hispanic patients with triple-negative subtype were significantly less likely to survive (*p* < 0.05).

**Table 6:** OS at 7.5 years for race and ethnicity grouped by tumor subtypes

	ER−/HER2+	ER+/HER2+	ER+/HER2−	ER−/HER2− (TN)
OS				
White	14 (100%)	32 (89%)	41 (91%)	30 (77%)
Black	29 (81%)	31 (89%)	44 (85%)	64 (65%)

	ER−/HER2+	ER+/HER2+	ER+/HER2−	ER−/HER2− (TN)
OS				
Hispanic	17 (81%)	25 (93%)	39 (85%)	30 (71%)
Non-Hispanic	32 (86%)	44 (88%)	59 (88%)	74 (68%)^b^

^a^Indicates *p* < 0.05 between race or ethnicity

^b^Indicates *p* < 0.05 different from ER−/HER2+ (*N* = 475)

Table 7 shows the OS odds ratios for demographics, tumor subtypes, pCR, income quintile, and insurance status. Blacks and Asians had worse survival ORs compared to Whites (*p* < 0.05). Triple negative had worse OR compared to ER−/HER2+ (*p* = 0.025). The other subtypes showed no worse odds of OS compared to ER−/HER2+ (*p* > 0.05). OS was not associated with income quintiles, but patients on Medicaid and Medicare had worse ORs compared to those on private insurance. As noted above patients on Medicare were significantly older (*p* < 0.001, ANOVA).

**Table 7:** Odds ratios for OS at 7.5 years for demographics, tumor subtypes, pCR, income quintile, and insurance status (*N* = 433 out of 475)

	OR	2.5%	97.5%	*p* value
Race				
Black versus non-Hispanic White (ref)	0.254	0.088	0.734	0.011
Asian versus non-Hispanic White (ref)	0.314	0.099	0.813	0.019
Ethnicity				
Hispanics	1.182	0.701	1.993	0.531
Subtypes (ER− and HER2+ as reference)				
Triple negative	0.406	0.184	0.895	0.025
ER+ and HER2+	1.584	0.571	4.395	0.377
ER+ and HER2−	1.200	0.490	2.936	0.690
Incomes (5th quintile (highest) as reference)				
4th	0.599	0.162	2.222	0.444
3rd	0.377	0.100	1.417	0.149
2nd	0.308	0.096	0.991	0.05
1st	0.514	0.148	1.787	0.296
Insurance (private as reference)				
Medicaid	0.281	0.116	0.681	0.005
Medicare	0.121	0.046	0.320	< 0.0001

Table 8 summarizes the results of the ML models. The top 10 predictors were similar for all 4 models, with high accuracy, specificity, and accuracy. AUC ranged from 0.84 to 0.85. Note that these models which included MRI data performed better than those that did not include MRI data.

**Table 8:** Multivariate results of top predictors of OS for all 4 models utilizing top 10 predictors including MRI data (*N* = 240)

	Predictors	Acc	Spec	Sens	AUC
Neural network	Tumor size, T-stage, nipple involvement, N stage, pCR, triple negative, skin involvement, lymph node involvement, ER+/HER2, pectoralis muscle involvement	0.947 ± 0.022	0.986 ± 0.019	0.062 ± 0.076	0.840 ± 0.117
Random forest	Tumor size, T-stage, N stage, pCR, triple negative, age, nipple involvement, skin involvement, lymph node involvement, ER+/HER2	0.915 ± 0.035	0.932 ± 0.025	0.667 ± 0.209	0.830 ± 0.045
Logistic regression	Tumor size, T-stage, pCR, N stage, triple negative, nipple involvement, skin involvement, age, ER+/HER2, pectoralis muscle involvement	0.898 ± 0.055	0.985 ± 0.012	0.310 ± 0.284	0.850 ± 0.098
Gradient boosted regression	Tumor size, T-stage, pCR, triple negative, nipple involvement, N stage, skin involvement, lymph node involvement, age, ER+/HER2	0.869 ± 0.306	0.953 ± 0.031	0.244 ± 0.167	0.841 ± 0.118

## Discussion

This study employed multiple machine learning models to predict pCR and OS using patient demographics, clinicopathologic tumor characteristics, and MRI radiology report data from a diverse racial and ethnic patient population, many of whom had lower socioeconomic status. The major findings are: (1) pCR is associated with tumor stage, and tumor size and BPE, but not race, ethnicity, income quintile, and insurance status, (2) ER−/HER2+ has the highest pCR rate, followed by triple negative, ER+/HER2+ and ER+/HER2−, (3) all 4 machine learning models consistently rank ER+/HER2−, ER−/HER2+, radiographic tumor size, and BPE as top predictors of pCR (AUC = 0.74–0.76), (4) OS is associated with pCR status, tumor subtype, tumor stage, some MRI data, and insurance status, race and ethnicity. All 4 models consistently rank ER+/HER2−, ER−/HER2+, radiographic tumor size, and BPE as top predictors of OS (AUC = 0.83–0.84), (5) pCR, and certain tumor subtype, and private insurance status are associated with higher survival probability, (6) when grouping race and ethnicity by tumor subtypes, neither pCR nor OS outcomes was different due to race and ethnicity for each tumor subtype.

### pCR

Studies evaluating associations between pCR and race and ethnicity have reported conflicting results [24–39]. Studies utilizing data from the National Cancer Database (NCDB), have demonstrated lower pCR rates in Black women with triple negative or HER2+ disease. [27, 34, 40]. However, as the NCDB does not capture specifics of treatment, these findings may reflect disparities in access to or quality of care between Black and white patients. Retrospective evaluations of patients treated at single institutions, or treated on multi-institution clinical trials, who likely received more uniform care, have shown differing results, with some demonstrating no association between race and pCR [25, 26, 33, 38], and others showing poorer outcomes for Black women [35]. Differences in treatment could account for the differing outcomes in the various studies. Knisley et al. found that white women more likely completed the recommended course of NAC treatment than did African American women [41]. Two studies by Griggs et al. demonstrated that black women with early stage breast cancer are more likely to receive substandard dose of chemotherapy, lower relative dose intensity, dose reductions in a treatment cycle, and delay in start of chemotherapy relative to white women [42, 43]. Black patients experience a higher rate of cardiotoxicity compared to white patients with adjuvant HER2-targeted therapy, resulting in incomplete treatment [44]. Enhanced cardiac surveillance, cardioprotective strategies, and early referral to cardiology when appropriate may be of benefit [44]. A prospective study where Black breast cancer patients received the same care as white breast cancer patients demonstrated equivalent disease specific survival, illustrating that equal outcomes between Blacks and whites are achievable when treatment disparities are eliminated [45].

We found no evidence that pCR was associated with race or ethnicity per se in our healthcare system, after adjusting for covariates such as tumor subtypes. Instead, our data showed strong association between tumor subtypes and pCR, consistent with multiple other prior studies [46–52], with ER−/HER2+ tumors exhibiting highest pCR rates, followed by triple negative, ER+/HER2+ and ER+/HER2−. When grouping race and ethnicity by tumor subtypes, Blacks showed a higher composition of triple negative as expected but there were no differences in pCR due to race for each tumor subtypes. Our results might reflect relatively equal healthcare access and treatment for breast cancer across the spectrum of racial groups in our healthcare system. Larger multi-center studies are needed to confirm these findings. Tumor differentiation, as expressed by Nottingham grade, was not predictive of pCR. Previous studies have reported both with and without association between tumor grade and pCR [47–52]. Insurance status and income quintile were not significantly associated with pCR.

Tumor biology consistently emerges as a factor linked to pCR [53, 54]. Previously reported racial disparities in survival may be due to factors which are potentially inter-related and would therefore be difficult to isolate from one another, such as socioeconomic differences, differences in insurance, and differences in treatment. Facilitating health care access and standardizing treatments across racial groups would help in eliminating such disparities.

MRI tumor size and BPE at presentation were significant predictors of pCR. Smaller tumor size was associated with higher pCR rates, suggesting that early detection and intervention may contribute to improved treatment outcomes. This is in keeping with several other studies [56–58]. Qian et al. found lower T scores and smaller tumor size correlated with higher pCR rates [54]. Goorts et al. reported lower T stages had significantly higher pCR and found that cT3/cT4 were independent risk factors for decreased pCR [55]. Another study found tumor size greater than 5 cm had a lower likelihood of pCR and that receptor status had the greatest impact on pCR, though both receptor status and tumor size were important [56]. They also saw no significant relationship between tumor size and receptor status [56]. Of note, in machine learning analysis, tumor size was consistently predictive for pCR but tumor stage was less predictive. This discrepancy could be because between tumor size by longest diameter was obtained from radiology report, which was a coarse measurement by a radiologist in a clinical setting. Mild BPE at presentation also correlated with higher pCR rates, indicating that the absence of extensive benign tissue may facilitate treatment response. These findings underscore the potential importance of imaging features in predicting treatment outcomes. There is no consensus in the literature on the association between BPE and pCR. One study showed BPE may be associated with pCR in limited circumstances, and another study showed BPE was associated with lower pCR in HR+/HER2− breast cancer patients [24, 31]. While tumor subtypes are invariant for each patient, tumor size, BPE and other imaging characteristics are modulated by treatment across time; thus, the temporal evolution of imaging characteristics can provide additional and useful data to predict outcomes.

Four machine learning models consistently identified and ranked ER+/HER2−, ER−/HER2+, tumor size, and BPE as the top predictors of pCR, followed by Nottingham grade, nodal and tumor staging. This convergence among the models reinforces the significance of these variables in predicting treatment response. Given the small sample size, there were not sufficient data to vigorously test which machine learning model was superior. Although many prior studies have reported the predictive value of tumor subtypes for pCR, the accuracy of these predictions based on tumor subtypes alone ranged from modest to moderate [57, 58]. Our patient cohort is unique due to its diversity, lower socioeconomic status, and a higher prevalence of triple-negative cancer. Our institution is a National Cancer Institute designated cancer center university hospital where patients had access to clinical trials and state-of-the-art treatment, which may also explain why race was not a factor in pCR.

Finally, we noted that the addition of MRI data to the model outperformed prediction of pCR without using MRI data. Higher AUC was similarly achieved in a prior study by combining clinical and imaging data in predicting pCR with ML from a public dataset [37, 59]. Accurately determining which breast cancer patients are likely to respond to neoadjuvant chemotherapy can aid in targeting type and dosing of medications to likely responders while minimizing unnecessary treatment to non-responders to maximize favorable outcomes.

### Overall survival

OS have also been reported to be worse in minority and underserved populations [60, 61]. Reeder-Hayes et al. after adjusting for age, comorbidities, disease characteristics including type of locoregional therapy, and neighborhood poverty, found that Black women were 25% less likely to receive monoclonal antibody treatment than white women among Medicare beneficiaries with stage I to III HER2+ diagnosed in 2010 and 2011 [62]. We found significant differences in OS due to race but not ethnicity by logistic regression analysis. OS was also significantly associated with tumor subtypes, with triple-negative subtype exhibiting worse OS, emphasizing the aggressive nature of this subtype and the need for targeted treatment approaches. In addition to race and molecular subtypes, access to care and other factors could contribute to different outcomes. OS was also significantly associated with pCR, consistent with multiple clinical trials demonstrating improved breast cancer outcomes in patients who achieve pCR, with prognostic value greatest for aggressive tumor subtypes [47–52, 63].

When grouping race and ethnicity by tumor subtypes, we found no differences in OS that were due to race and ethnicity. This is consistent with the observation that OS was not associated with race after stratified by pCR (Fig. 1), corroborating that tumor subtypes play a more important role in pCR, than race and ethnicity per se.

Patients on Medicare and Medicaid had worse OS outcomes than those on private insurance. Medicare patients were generally older, which could have contributed to worse outcomes. This is in accordance with prior studies that have shown disparities in outcomes based on insurance. Avanian et al. showed that women without insurance or with Medicaid had worse overall survival with 49% and 40% higher risk of death, respectively [64]. Underinsured women may not be able to access ancillary services that have been shown to improve breast cancer outcomes, such as exercise programs, nutrition courses, and psychotherapy [65–67]. Additionally, insurance may be reflective of socioeconomic factors that may influence both oncologic and non-oncologic outcomes, such as medical insight, income, healthcare access, and nutritional status [68–70].

Our data showed OS was not associated with income quintiles. Several studies have reported associations between expansion of Medicaid coverage and improved survival in cancer patients, and other studies have found that greater levels of financial toxicity predict for poorer oncologic outcomes [71–73]. Association of income inequalities with increased mortality has been noted in other studies [74, 75]. One study revealed excess mortality hazard for breast cancer to be lower for individuals in higher income quintiles in their study population after adjusting for age, education, and occupation [76].

Machine learning models consistently identified and ranked tumor size, nodal stage, and pCR as the top predictors of OS, following by some tumor subtypes. A meta-analysis of 21 studies showed that the number of circulating tumor cells detected before NAC in early breast cancer patients was markedly associated with tumor size and had a detrimental effect on overall survival and on distant disease-free survival but was not associated with receptor status or pCR [77]. This suggests that the tumor size and tumor microenvironment exert a significant effect on outcomes independent of receptor status and pCR [77].

The addition of MRI data outperformed predictions of OS without MRI data. This highlights the potential of MRI data as non-invasive tools to support treatment decision-making and improve prognostic accuracy. Note that some tumor subtypes and tumor size (but not clinical staging) were top independent predictors of pCR, whereas tumor size and clinical stage (but not tumor subtypes) were top independent predictors of OS. These could be due to the potential interaction among different variables. Note that pCR is not the top predictor of OS (only among top 3 to 5, depending on models). Machine learning approaches offer a means to account for covariates and interactions among variables.

### Limitations

There are several limitations. Our sample sizes are small when stratified further by molecular subtypes; and thus, those findings need to be interpreted with caution. The sample size for MRI radiology reports was small. We utilized MRI radiology reports as inputs rather than the actual images themselves. Future investigations could incorporate deep learning analysis of breast cancer images, which may further improve prediction accuracy and provide additional insights. Our cohort had small proportion of Caucasians which could contribute to difference in findings with literature. Our cohort had small proportion of Caucasians which could contribute to difference in findings with literature.

Income data were based on zip codes and individual patients’ status might be different for those based on zip codes. Some patients might have multiple insurance and we only used the primary insurance in our analysis. Attrition rate due to relocation could result in missing mortality data; and thus, it is possible some patients might have expired that were not accounted for.

Changes in neoadjuvant therapies and post neoadjuvant treatment may impact the validity of predictive models but they were not accounted for in predictive models. For example, the addition of immunotherapy to neoadjuvant chemotherapy for triple-negative breast cancer was not the standard of care at the time that our patient cohort was treated; therefore, our findings may not be generalizable to patients treated with immunotherapy. Axillary lymph node data have been used to predict PCR and OS [78–80].

## Conclusion

This study employed multiple machine learning models to predict pCR and survival in a racially and ethnically diverse patient population from an underserved inner-city community. Incorporating imaging data alongside tumor subtypes enhances the accuracy of predictions. Race, but not ethnicity, and insurance status, but not incomes, were associated with worse survival. These findings have implications for personalized cancer treatment strategies and emphasize the need for further research in cancer treatment outcomes with respective to health disparity.

### Supplementary Information

**Additional file 1: Table S1.** AUCs for all four univariate models across all 4 tumor types (output variable is pCR) (N = 240).

## Data Availability

Data are available on reasonable request. De-identified data used during the study are available from the corresponding author upon reasonable request.
