# Supplementary material for: Machine learning prediction of pathological complete response and overall survival of breast cancer patients in an underserved inner-city population
Source: Breast Cancer Res. 2024 Jan 10;26:7. doi: 10.1186/s13058-023-01762-w (PMC10782738; doi:10.1186/s13058-023-01762-w)
Supplement: Supplementary file 1 — Additional file 1: Table S1. AUCs for all four univariate models across all 4 tumor types (output variable is pCR) (N = 240). [file 13058_2023_1762_MOESM1_ESM.docx]

**Table S1.** AUCs for all four univariate models across all 4 tumor types (output variable is pCR) (N=240).

|  | ER+/HER2+ | | | | ER+/HER2- | | | | ER-/HER2+ | | | | Triple Negative | | | | |
| --- | --- | --- | --- | --- | --- | --- | --- | --- | --- | --- | --- | --- | --- | --- | --- | --- | --- |
| Model | NN | RF | Logistic | GBR | NN | RF | LR | GBR | NN | RF | Logistic | GBR | NN | RF | LR | GBR |  |
| BPE | 0.6262 | 0.6262 | 0.6262 | 0.5769 | 0.6250 | 0.6250 | 0.6250 | 0.6198 | 0.5854 | 0.5854 | 0.5140 | 0.5357 | 0.5897 | 0.5897 | 0.5897 | 0.5848 |  |
| Pre NAT NHS | 0.5244 | 0.5244 | 0.5075 | 0.5085 | 0.6558 | 0.6558 | 0.3442 | 0.6501 | 0.5983 | 0.5983 | 0.5983 | 0.5983 | 0.5962 | 0.5962 | 0.5397 | 0.5680 |  |
| Age | 0.5175 | 0.6825 | 0.5175 | 0.5522 | 0.6092 | 0.7651 | 0.6092 | 0.6627 | 0.6193 | 0.7127 | 0.6193 | 0.5833 | 0.5000 | 0.6235 | 0.5338 | 0.5618 |  |
| Tumor Size | 0.7015 | 0.6221 | 0.6221 | 0.6618 | 0.5546 | 0.5718 | 0.4454 | 0.5632 | 0.6986 | 0.6962 | 0.5766 | 0.6364 | 0.6524 | 0.6313 | 0.4884 | 0.5598 |  |
| N Stage | 0.5209 | 0.5866 | 0.5194 | 0.5201 | 0.5817 | 0.6543 | 0.5817 | 0.618 | 0.6061 | 0.6061 | 0.6061 | 0.5904 | 0.5328 | 0.5328 | 0.5261 | 0.5267 |  |
| Pectoralis Involvement  Involvement | 0.5750 | 0.5750 | 0.5750 | 0.5750 | 0.5692 | 0.5692 | 0.5692 | 0.5692 | 0.6429 | 0.6429 | 0.6429 | 0.6429 | 0.5489 | 0.5489 | 0.5489 | 0.5323 |  |
| Ethnicity | 0.5515 | 0.5515 | 0.5515 | 0.5515 | 0.6207 | 0.6207 | 0.6207 | 0.6207 | 0.5044 | 0.4956 | 0.5044 | 0.5044 | 0.5232 | 0.5232 | 0.5232 | 0.5232 |  |
| Multicentric | 0.5587 | 0.5587 | 0.4913 | 0.525 | 0.5628 | 0.5628 | 0.5628 | 0.5628 | 0.5804 | 0.5804 | 0.4911 | 0.5357 | 0.5416 | 0.5416 | 0.5416 | 0.5323 |  |
| Multifocal | 0.5663 | 0.5663 | 0.5663 | 0.525 | 0.5949 | 0.5949 | 0.5949 | 0.5936 | 0.4970 | 0.5030 | 0.5030 | 0.5029 | 0.5921 | 0.5921 | 0.5599 | 0.5161 |  |
| Skin Involvement | 0.5481 | 0.5481 | 0.5481 | 0.5481 | 0.5469 | 0.5365 | 0.4688 | 0.5339 | 0.6310 | 0.6310 | 0.6310 | 0.6309 | 0.5908 | 0.5864 | 0.5864 | 0.5864 |  |
| Nipple  Involvement | 0.5394 | 0.5394 | 0.5394 | 0.525 | 0.6000 | 0.5795 | 0.5692 | 0.5744 | 0.5774 | 0.5595 | 0.5476 | 0.5536 | 0.5984 | 0.5984 | 0.5662 | 0.5694 |  |
| Race | 0.5448 | 0.5448 | 0.4843 | 0.5302 | 0.5856 | 0.6001 | 0.5856 | 0.5837 | 0.5671 | 0.5741 | 0.5671 | 0.5588 | 0.5254 | 0.5324 | 0.5254 | 0.5174 |  |
| Satellite Lesions | 0.5375 | 0.5375 | 0.5375 | 0.5125 | 0.5628 | 0.5974 | 0.5974 | 0.5949 | 0.6057 | 0.6057 | 0.5342 | 0.5357 | 0.5869 | 0.5869 | 0.4929 | 0.5399 |  |
| Chest Wall  Involvement | 0.5125 | 0.5125 | 0.5125 | 0.5125 | 0.5821 | 0.5692 | 0.5667 | 0.5679 | 0.5357 | 0.5357 | 0.5357 | 0.5357 | 0.5161 | 0.5161 | 0.5161 | 0.5161 |  |
| Nodal Involvement | 0.5694 | 0.5694 | 0.5694 | 0.5366 | 0.5628 | 0.5628 | 0.4372 | 0.5628 | 0.6250 | 0.6250 | 0.6250 | 0.5714 | 0.5410 | 0.5115 | 0.5115 | 0.5262 |  |
| T Stage | 0.4955 | 0.5591 | 0.5000 | 0.5295 | 0.5801 | 0.5801 | 0.5801 | 0.5805 | 0.4934 | 0.5311 | 0.4934 | 0.5123 | 0.6107 | 0.6107 | 0.6107 | 0.6122 |  |
